# Assessing clinical research coordinator knowledge of good clinical practice: An evaluation of the state of the art and a test validation study

**DOI:** 10.1017/cts.2019.440

**Published:** 2020-02-19

**Authors:** James M. DuBois, Jessica T. Mozersky, Alison L. Antes, Kari Baldwin, Michelle Jenkerson

**Affiliations:** Washington University School of Medicine, St. Louis, MO, USA

**Keywords:** Good clinical practice, clinical research coordinators, clinical trials, testing, validation, measurement, knowledge

## Abstract

This paper describes the development and validation of a new 32-item test of knowledge of good clinical practice (GCP) administered to 625 clinical research coordinators. GCP training is mandated by study sponsors including the US National Institutes of Health. The effectiveness of training is rarely assessed, and the lack of validated tests is an obstacle to assessment. The GCP knowledge test was developed following evaluation of two existing widely used GCP tests to ensure it accurately reflects the content of current training. The final GCP knowledge test demonstrated good reliability (*α* = 0.69). It is a valid and reliable instrument for measuring knowledge of GCP. The test will be useful in assessing the effectiveness of GCP training programs as well as individuals’ mastery of GCP content.

## Introduction

International standards for good clinical practice (GCP) foster participant safety, regulatory compliance, and scientific rigor [1,2]. The importance of clear standards for GCP has increased as clinical trials have grown increasingly complex and international in scope. GCP training is a key component of ensuring that clinical trialists are prepared to meet their professional obligations. Recently, the US National Institutes of Health (NIH) mandated that all clinical trial investigators and staff complete training in GCP and refresh training every 3 years [3]. A growing number of professional societies, funding agencies, and clinical trial sponsors now offer GCP training programs [4]. In 2014 and 2017, expert consensus committees adopted eight core competencies for GCP training programs, each with specific sub-competencies [4–6]. However, GCP training programs are currently highly variable in format (including diverse online and face-to-face formats), duration (from 45 minutes to lengthy certification programs), and scope of content. It is unclear how well curricula cover the proposed eight competencies [5,6].

Training programs can be expensive and time-consuming [5]. Evidence indicates that training programs in research ethics differ significantly in effectiveness: While programs in general are not associated with any positive outcomes [7,8], some programs are associated with improved knowledge and decision-making skills [9]. It is therefore essential to assess training programs to determine whether they are effective [10,11]. Moreover, assessment enables programs to determine when an individual has adequately mastered important content [11]. For example, online training programs frequently require learners to score 80% correct on tests of knowledge.

At present, there are no validated measures for assessing knowledge of GCP.

This project had two primary aims:

1. Evaluate the quality and scope of current GCP test items that are in use by leading training programs.
2. Develop and validate a multiple-choice (MC) test of GCP knowledge.

## Materials and Methods

Our approach involved evaluating existing GCP knowledge test items, writing new GCP knowledge MC test items, administering the GCP test to a sample of clinical research coordinators (CRCs), and statistically analyzing data to create a final test comprised of items that met our psychometric criteria. The item-writing team included two individuals with PhDs in psychology and experience developing and validating assessment instruments (AA and JD), and two individuals with experience as CRCs, training CRCs, and providing consultations on regulatory matters (MJ and JTM) within the Washington University in St. Louis Clinical and Translational Science Award (CTSA) program.

### Item Evaluation and Development Methods

**Fig. 1.**
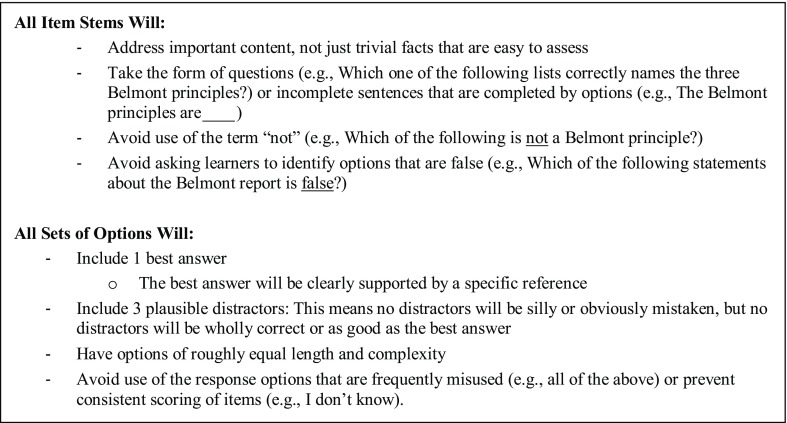
Criteria for evaluating and writing multiple-choice (MC) test items+

We examined a total of 136 MC items from 2 leading online GCP training programs (CITI Program and the Association for Clinical Research Professionals). We adopted the criteria listed in Fig. 1 for evaluating, and eventually re-writing, MC test items. These criteria are based on the work of Haladyna [12], who conducted a systematic review and evaluation of criteria for writing MC items, and Case and Swan, who developed test-writing criteria for the National Board of Medical Examiners [13].

Using these criteria, we evaluated all items for importance (significance to GCP) and appropriate structure. All items were independently reviewed by the two individuals who have worked as CRCs and are leaders within the Washington University in St. Louis Clinical and CTSA program’s regulatory knowledge and support core. Importance was rated on a 3-point scale: important (3), somewhat important (2), or unimportant (1). Rating discrepancies were resolved through discussion and consensus.

The two raters also reviewed the 136 GCP MC knowledge items to categorize them into 1 of the 8 core competencies of GCP (2014 version) [6]. Rating discrepancies were discussed with the principal investigator (PI; JMD) and resolved through consensus after reviewing which specific sub-competencies were subsumed under each of the eight competencies.

Following evaluation of the 136 existing items, we wrote a new test covering the important content addressed by existing items. We wanted a test that would be brief, valid, and demonstrate acceptable reliability. We wanted to ensure our test accurately reflected the content of current training, rather than proposed new content such as trial design, so that the test could be used to assess CRCs’ mastery of training material. Therefore, we wrote 35 new items covering the 4 competency domains regularly covered in GCP training for CRCs: clinical trial operations, study site management, ethical and participant safety considerations, and data management and informatics. The material commonly covered under the competency of “medicine development” was considered of greater significance to PIs and sponsors.

### Cognitive Interviews

Prior to launching our validation survey, we conducted online cognitive interviews [14]. Thirteen experienced CRCs each reviewed approximately 25% of our items; all items were reviewed by at least three CRCs. CRCs were asked if the instructions were clear, and for each item:

1. Which do you think is the correct response?
2. Did you have a difficult time responding to the item? If yes, why?
3. Do you think this item is unfair or inappropriate to ask a CRC working in the USA?
4. Option __ is correct: Do you have any reason to believe this option is incorrect?

All comments were reviewed by the entire item-writing team (JTM, JDM, ALA). No items were dropped; five items were revised to improve clarity and ensure that only one response was best.

### Survey Methods and Participants

This study was approved by the Human Research Protections Office of Washington University in St. Louis. All participants were presented with a study information sheet to read prior to proceeding to the survey.

The GCP knowledge test was administered with several other measures [15] using the Qualtrics survey platform. We enrolled participants from three research-intensive medical centers in the USA, which have NIH CTSAs. Collaborating institutions preferred not to share the names and email addresses of their employees; therefore, representatives at CTSAs of each collaborating institution distributed anonymous survey links. Participants received three follow-up email reminders.

Across all three institutions, 2415 emails were sent inviting clinical research staff to participate. Twenty-two percent of respondents were ineligible to participate because they were not CRCs; thus, we estimate that we invited 1884 eligible individuals. At Institution I, 286 CRCs completed the test; at Institution II, 185; and at Institution III, 157 for a total of 628 participants. We estimate that 33% of eligible individuals completed the survey.

We examined participant performance to determine if all cases should be retained. Three of 628 were dropped because we believed their data were invalid: They were outliers on Fig. 2 (more than 1.5 times the interquartile range below the lower quartile with scores of 37% or lower) and spent very little time on the entire survey (<10 minutes versus a sample median of 39 minutes).

### Data Analysis Methods

With the remaining sample of 625, we calculated descriptive statistics (score range, median, mean, and standard deviation) to characterize performance of the sample. We calculated Cronbach’s alpha as a measure of internal consistency reliability. To establish convergent validity, we used Pearson’s *r* and a *t*-test for independent samples to examine the relationship of test scores with two variables we hypothesized to be positively related with higher scores (years of experience and certification as a CRC).

## Results

### Evaluation of the 136 Existing Items Used by Training Programs

The 136 existing items that we evaluated prior to writing original items addressed five of eight core areas of GCP: clinical trial operations (*n* = 66); study and site management (*n* = 27); ethical and participant safety considerations (*n* = 21); medicines development and regulation (renamed “investigational products development and regulation” in 2017) (*n* = 10); and data management and informatics (*n* = 7). Five items straddled two core areas. No items addressed the following three domains: scientific concepts and research design; leadership and professionalism (renamed “leadership, professionalism, and team science” in 2017); and communication and teamwork.

Of 136 total items, 5 items were rated as “unimportant” and 30 as “somewhat important”; 101 items were deemed “important” for CRCs and were further evaluated. Of these 101 items, 46 violated at least one further item-writing criteria (see Fig. 1).

### Demographics and Evaluation of New GCP Knowledge Test Items

Table 1 presents demographic data for our 625 participants.

**Table 1.** Demographics (N = 625)

	Frequency	%
Age		
18–25	76	12
26–34	213	34
35–44	147	24
45–54	104	17
55–64	76	12
65+	9	1
Gender		
Female	546	87
Male	79	13
Race		
White	477	76
African American	73	12
Asian	47	8
American Indian or Alaska Native	2	0.3
Multiple racial categories	21	3
Other	5	1
Certified as a clinical research coordinator (yes)		
	178	29

	Mean	SD
Years of experience as clinical research coordinator		
	6.92	6.63

Table 2 presents the difficulty factor, corrected item-total correlations, and the point-biserial correlations for each of the 35 new GCP knowledge test items. Three items had negative corrected item-total correlations and negative or non-significant point-biserial correlations, and accordingly were dropped. The following statistical analyses were conducted with the final 32-item version of the GCP knowledge test.

**Table 2.** Statistical evaluation of 35-item version (N = 625)

Item #	Number correct	Difficulty factor	Corrected item-total correlation	Point-biserial correlation
GCPMC1	221	0.35	0.208	0.328**
GCPMC2	574	0.92	0.043	0.116**
GCPMC3	604	0.97	0.162	0.208**
GCPMC4	598	0.96	0.263	0.313**
GCPMC5	533	0.85	0.205	0.294**
GCPMC6	581	0.93	0.264	0.327**
GCPMC7	355	0.57	0.411	0.518**
GCPMC8	609	0.97	0.195	0.235**
GCPMC9	584	0.93	0.198	0.261**
GCPMC10	419	0.67	0.182	0.302**
GCPMC11	515	0.82	0.247	0.341**
GCPMC12	591	0.95	0.168	0.226**
**GCPMC13**	**268**	**0.43**	**−0.056**	**0.076**
**GCPMC14**	**201**	**0.32**	**−0.056**	**0.068**
GCPMC15	510	0.82	0.201	0.299**
GCPMC16	376	0.60	0.092	0.220**
**GCPMC17**	**32**	**0.05**	**−0.096**	**−0.037**
GCPMC18	423	0.68	0.262	0.376**
GCPMC19	473	0.76	0.284	0.388**
GCPMC20	353	0.56	0.119	0.248**
GCPMC21	278	0.44	0.126	0.255**
GCPMC22	440	0.70	0.236	0.349**
GCPMC23	561	0.90	0.189	0.267**
GCPMC24	508	0.81	0.251	0.348**
GCPMC25	343	0.55	0.140	0.269**
GCPMC26	394	0.63	0.261	0.378**
GCPMC27	604	0.97	0.326	0.369**
GCPMC28	565	0.90	0.256	0.329**
GCPMC29	558	0.89	0.269	0.345**
GCPMC30	522	0.84	0.142	0.238**
GCPMC31	569	0.91	0.289	0.358**
GCPMC32	490	0.78	0.229	0.332**
GCPMC33	340	0.54	0.305	0.423**
GCPMC34	429	0.69	0.139	0.259**
GCPMC35	563	0.90	0.261	0.334**

GCPMC, good clinical practice multiple choice.

Bold items were discarded for negative or non-significant point-biserial correlations.

* *p* < 0.01.

** *p* < 0.001.

### Descriptive Statistics, Reliability, and Convergent Validity of 32-item GCP Knowledge Test

Our sample had a range from 7 (22% correct) to 32 (100% correct). The mean score was 24.77 out of 32 (77% correct) with a standard deviation of 3.77 and a median score of 25 (78% correct). The GCP knowledge test demonstrated adequate alpha reliability for a MC knowledge test (*α* = 0.69). (Supplementary Fig. 1 presents the distribution of test scores for our sample.)

The two convergent validity measures (years of experience as a CRC and formal degree or certification as a CRC – all levels combined) were significantly, positively related to GCP knowledge scores: years of experience (*r* = 0.34, *p* < 0.001) and certification (*t* = 5.75, *p* < 0.001).

## Discussion

The GCP knowledge test is a brief 32-item test of GCP knowledge that demonstrated good convergent validity and alpha reliability (*α* = 0.69) in a large sample (*N* = 625) of CRCs. It will be useful in assessing outcomes of training programs, as well as individual learners’ mastery of subject matter.

It is a strength that the test reflects the current content of courses as this enables it to be used to assess CRCs’ mastery of training material. However, there is a discrepancy between statements on the core curriculum for GCP and actual training programs. GCP training courses generally focus on the roles of CRCs rather than PIs. From this perspective, it may be reasonable that training programs emphasize clinical trial operations, study and site management, ethical and participant safety considerations, and data management and informatics. While all eight core competencies are relevant to GCP, the topics of scientific concepts and research design; leadership and professionalism; medicine development; and communication and teamwork may be more needed by PIs than CRCs, given the leadership and scientific roles played by PIs. Nevertheless, everyone on a clinical trial team, including CRCs, would benefit from basic knowledge in these areas, and as training programs evolve, so too must knowledge assessment tools.

Our evaluation of existing GCP knowledge items suggests that current GCP training programs are not doing an adequate job assessing learning outcomes. A majority of items (60%) failed one or more criteria for writing valid MC items. This is not surprising if the item development process was not guided by evidence-based item-writing criteria, cognitive interviewing, and psychometric evaluation of items.

### Limitations

We estimate that the survey had a participation rate of 33%. While such a response rate would be a limitation in some survey contexts, we aimed to generate (a) a purposive sample (CRCs with diverse levels of experience) that was (b) sufficiently large to conduct psychometric analysis of test properties; accordingly, our sample was satisfactory.

Nearly one-third of items had very low difficulty levels (>0.89) and were retained. The test has a mean score of 78% correct. From a psychometric point of view, an average correct rate of 50% is ideal, because it maximizes the ability of the test to reliably distinguish between those who know and do not know content. Despite this limitation, the test has acceptable reliability and excellent convergent validity with years of experience and certification as CRCs. Moreover, our sample consisted of people with a high level of expertise: they were all CRCs working in research-intensive academic medical centers with an average of 6.9 years of experience, thus one would expect scores to be skewed high. Lastly, and perhaps most importantly, research ethics training programs commonly require a score of 80% correct to pass the training. Thus, a test that prioritizes ideal psychometric properties by generating a 50% correct rate would cause frustration and not serve the field well.

Finally, test items have not been published with this article because making items, and especially answer keys, publicly available diminishes their usefulness for summative (as opposed to formative) assessment. Nevertheless, the authors will make the GCP knowledge test available to those who wish to assess GCP training programs or the knowledge of clinical research associates. The test and scoring guide may be requested on the research team’s test service webpage: https://bioethicsresearch.org/research-services/testing-services/.

## Supplementary material

For supplementary material accompanying this paper visit http://dx.doi.org/10.1017/cts.2019.440. click here to view supplementary material
