# Supplementary material for: Assessing clinical research coordinator knowledge of good clinical practice: An evaluation of the state of the art and a test validation study
Source: J Clin Transl Sci. 2020 Feb 19;4(2):141–5. doi: 10.1017/cts.2019.440 (PMC7159812; doi:10.1017/cts.2019.440)
Supplement: Supplementary file 1 [file S2059866119004400sup001.docx]

SUPPLEMENTAL MATERIALS

Supplementary Figure 1. Distribution of Good Clinical Practice Knowledge Test Scores
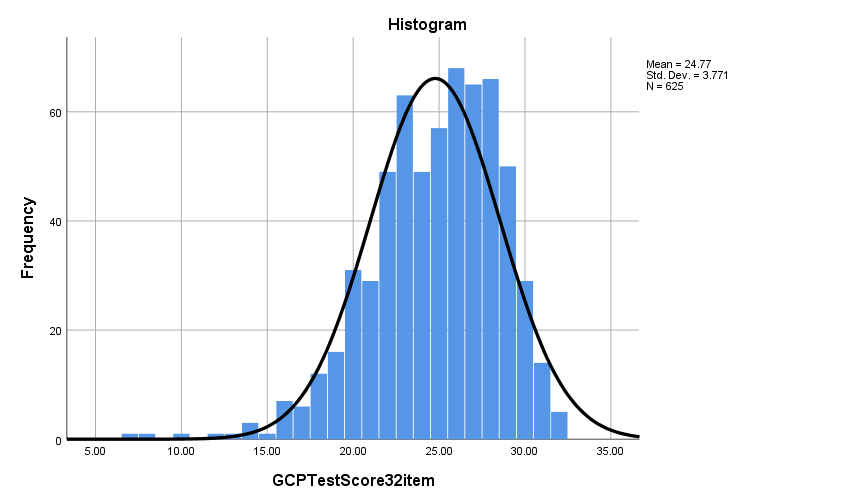


^a^

^a^

^a^ GCPTestScore32item stands for Good Clinical Practice Knowledge Test Score 32 item test
